# The Esophagus on Sight: Ultrasonography in Achalasia

**DOI:** 10.31662/jmaj.2018-0023

**Published:** 2018-09-28

**Authors:** Yoshito Nishimura, Yuusaku Sugihara, Fumio Otsuka

**Affiliations:** 1 Department of General Medicine, Okayama University Graduate School of Medicine, Dentistry and Pharmaceutical Sciences, Okayama, Japan

**Keywords:** Achalasia, Ultrasonography, Dysphagia

A 59-year-old man presented to the hospital because of dysphagia, nausea, and vomiting for a month. On transabdominal ultrasonography, regular hypoechoic wall thickening, massive dilatation, and narrowing of the distal segment of the esophagus were observed (Figure A). On neck ultrasonography, dilated esophagus was revealed (Figure B). A diagnosis of achalasia was established based on barium esophagography findings (Figure C). The patient underwent peroral endoscopic myotomy.

Achalasia is an esophageal motility disorder due to intramuscular neural plexus degeneration. Ultrasonography is a noninvasive method to detect esophageal dilatation^(1)^. It may be particularly effective in patients with dysphagia to differentiate achalasia from other disorders such as malignancies in which irregular wall thickening is noted^(2)^. Exposure to ionizing radiation can be avoided with ultrasonography, as against barium esophagography. In addition, ultrasonography is a less expensive and more direct maneuvering method. In cases involving dysphagia, ultrasonography should be considered as a valuable screening test.

**Figure.**
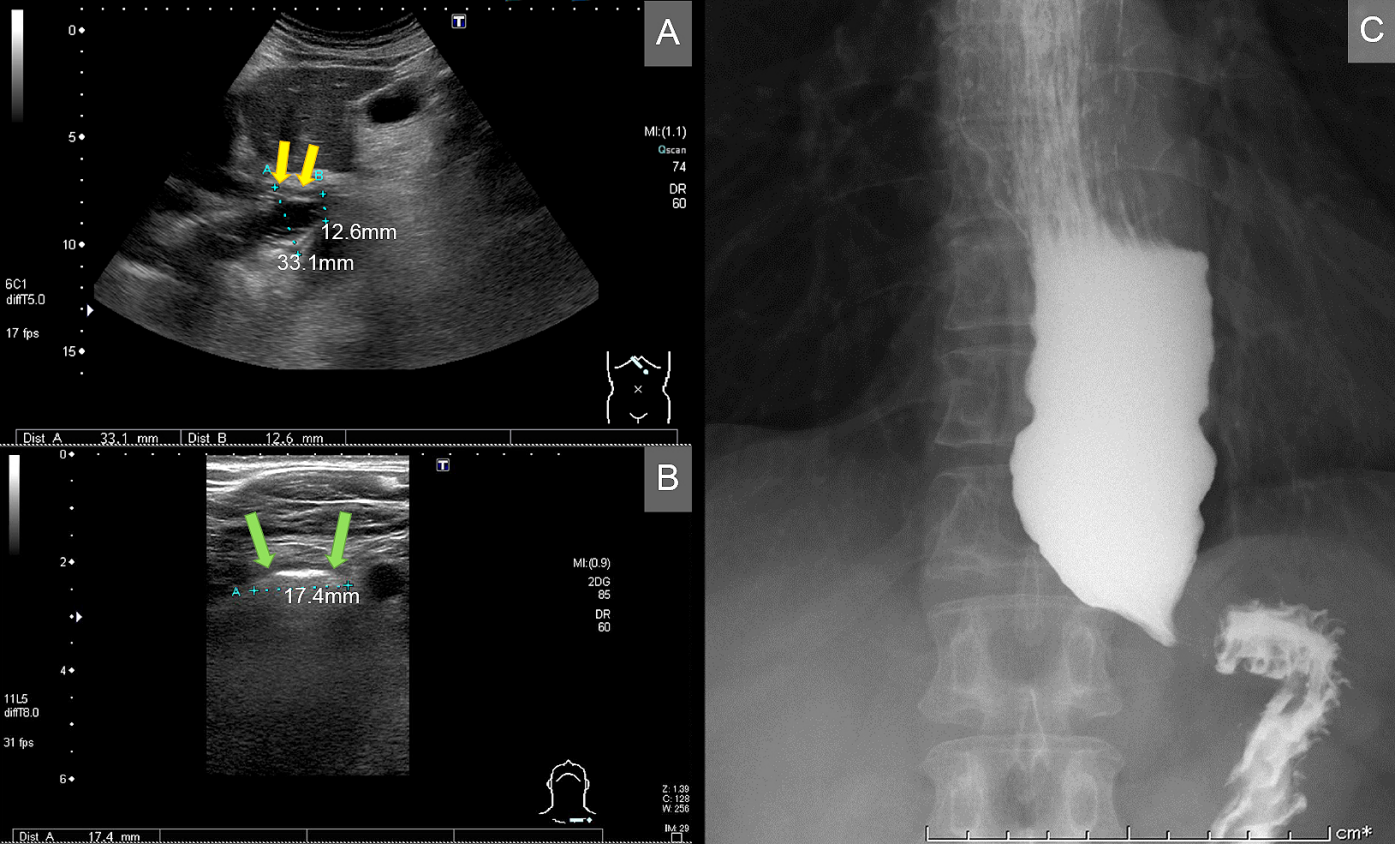
A transabdominal and neck ultrasonogram showing a dilated esophagus. (A) Significant dilatation and narrowing of the distal segment of the esophagus, with regular hypoechoic wall thickening (yellow arrow). (B) A neck ultrasonogram showing the dilated esophagus (green arrow). (C) A barium esophagogram showing the dilated esophagus, consistent with achalasia.

## Article Information

### Conflicts of Interest

None

### Author Contributions

YN wrote the manuscript. YS was an outpatient attending physician and edited the manuscript. FO supervised all procedures.

### Ethical Statement

Informed consent was obtained from the patient.
